# The complete mitogenome of the Critically Endangered smalltooth sand tiger shark, *Odontaspis ferox* (Lamniformes: Odontaspididae)

**DOI:** 10.1080/23802359.2020.1814886

**Published:** 2020-09-04

**Authors:** Noel Vella, Adriana Vella

**Affiliations:** Department of Biology, Conservation Biology Research Group, University of Malta, Msida, Malta

**Keywords:** Mitochondrial genome, phylogeny, Odontaspididae, *Odontaspis ferox*

## Abstract

Here, we report the first complete mitochondrial genome for the smalltooth sand tiger shark, *Odontaspis ferox* (Risso, 1810). The circular mitochondrial genome was found to be 16,682 bp in length and contains 37 genes, a control region and the replication origin of the L-strand (O_L_). The base composition of this mitogenome is 32.6% A, 23.3% C, 12.8% G, and 31.3% T. Phylogenetic analysis of Lamniformes indicates that *O. ferox* did not group with *Carcharias taurus* and so the taxonomic classification of Odontaspididae needs to be revised. This study promotes conservation genetics for this poorly studied shark species which is listed critically endangered in the Mediterranean Sea.

The smalltooth sand tiger shark, *Odontaspis ferox* (Risso, 1810), is one of the most poorly studied shark species (Fergusson et al. 2007), which is sparsely distributed in warm-temperate and tropical waters and is considered as uncommon given that it is rarely caught (Compagno 2002; Fergusson et al. 2007). Through the use of better data collection systems, recent new records of this species’ occurrence are giving a more comprehensive picture of its distribution (White 2007; Santander-Neto et al. 2011; Acuna-Marrero et al. 2013; Ritter and Compagno 2013; Long et al. 2014; Estupinan-Montano et al. 2016; Wellington et al. 2017). Moreover, on landing, this species is occasionally misidentified as *Hexanchus griseus*, given that both species have similar coloration (pers. obs.). Populations of *O. ferox* are declining and the species has been listed by IUCN as vulnerable on a global scale (Graham et al. 2016) and critically endangered at both European (Pollard et al. 2015) and Mediterranean level (Pollard et al. 2016). Consequently, it has been included in Annex II of the Specially Protected Areas and Biological Diversity (SPA/BD) Protocol (UNEP/MAP-SPA/RAC 2018) and in 2012 through the adoption of Recommendation GFCM36/2012/3, General Fisheries Commission for the Mediterranean prohibited the possession and commercialization of this species, while emphasizing on its possible unharmed release (FAO 2012). Subsequently, a number of Mediterranean countries have listed *O. ferox* as a protected species.

A 264 cm *O. ferox* male specimen was by-caught on 1 February 2011 through trawling activities in Maltese waters by local fishermen (36°5′3″N 14°4′43″E Central Mediterranean Sea). A tissue sample was collected from this specimen as part of fisheries landings sampling undertaken since 2002 by the Conservation Biology Research Group, University of Malta (CBRG-UM). The tissue sample collected for this species was stored at the Ichthyological Collection of the CBRG-UM (Ofer002-110201001) and has already contributed to DNA barcoding of the species (Vella et al. 2017). The total genomic DNA was extracted using GF-1 DNA Extraction Kit (Vivantis Technologies, Subang Jaya, Malaysia). A DNA library of the whole genome was constructed and next-generation sequencing reads were generated through Illumina HiSeqX using 2 × 150 bp end reads (Illumina, San Diego, CA). Sequences were paired, trimmed at Q ≥ Q30 and reads shorter that 100 nucleotides were discarded. The final data set was de novo assembled using Geneious R10 (Kearse et al. 2012). NCBI ORF Finder (https://www.ncbi.nlm.nih.gov/orffinder/) was used to identify PCGs, which were subsequently checked for start and stop codons. The tRNA genes were identified through their secondary structures using tRNAscan-SE v2.0 (Chan and Lowe 2019). This newly annotated genome was validated against the mitogenome of other Lamniformes species.

The complete mitogenome for this species is 16,682 bp long (GenBank accession: MT702386) and contains 13 PCGs, two rRNA genes, 22 tRNA genes, and two non-coding regions (control region and O_L_). The gene order followed the typical vertebrate order (Satoh et al. 2016), that is most of the mitochondrial genes are encoded on the H-strand, except one PCG (ND6), eight tRNA genes (Gln, Ala, Asn, Cys, Tyr, Ser, Glu, Pro) and the O_L_ which are encoded on the L-strand. The PCGs range between 168 bp (ATP8) and 1830 bp (ND5) encoding for a total of 3798 amino acids. All PCGs utilize ATG as their start codon except COX1 which uses GTG. The most common stop codon is TAA, while ND6 uses AGG and four genes (COX2, ND3, ND4, and cytB) use T––. The length of the 22 tRNA genes range from 67 bp (Ser^AGY^, Cys) to 75 bp (Leu^UUR^). All tRNA genes produced the expected cloverleaf structure except for Ser^AGY^ that has a missing dihydrouridine arm, typical for most vertebrate species (Satoh et al. 2016).

The currently sequenced mitogenome was aligned with that of 13 other Lamniformes using ClustalW (Thompson et al. 1994), while a phylogenetic tree, excluding the control region, was constructed using Bayesian Inference analysis through Mr Bayes v3.2.6 (Huelsenbeck and Ronquist 2001) (Figure 1) using GTR G + I substitution model as determined by jModelTest v2.1.7 (Darriba et al. 2012). This analysis did not group *O. ferox* with *Carcharias taurus* even though taxonomic keys place them both in Odontaspididae (Compagno 2002). Therefore, current results corroborate a number of morphological studies (Shimada 2005; Shimada et al. 2009) and molecular phylogenetic studies using smaller DNA sequences (Martin et al. 2002; Human et al. 2006; Velez-Zuazo and Agnarsson 2011; Naylor et al. 2012), which show that Odontaspididae is not monophyletic. This outcome indicates that the taxonomic classification of the two species that compose the family Odontaspididae needs to be revised. The genetic resources made available by this study on *O. ferox* aid future research into the genetics and evolution of its populations. It also promotes knowledge and research to better understand this species’ taxonomy to target effective measures toward its urgent conservation needs.

**Figure 1.**
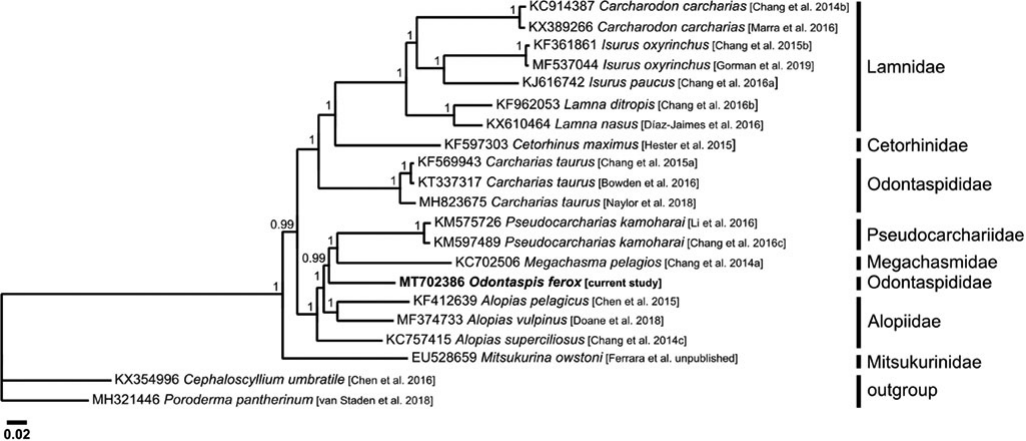
Bayesian inference based phylogeny depicting the mitogenomic relationship between 14 Lamniformes species using two Carcharhiniformes species as outgroup as inferred from their complete mitogenomes (excluding the control region). Each label includes the GenBank accession number, species, reference and respective family, while numbers at the nodes indicate the posterior probability values. This analysis used 5 × 10^6^ generations, a sample frequency every 1000 generations and a burn-in of 25%. The mean standard deviation of split frequencies was <0.001.

## Data Availability

The data that support the findings of this study are openly available in GenBank (accession no. MT702386) at https://www.ncbi.nlm.nih.gov/genbank/.
